# Virulence profile of different phylogenetic groups of locally isolated community acquired uropathogenic *E. coli* from Faisalabad region of Pakistan

**DOI:** 10.1186/1476-0711-11-23

**Published:** 2012-08-06

**Authors:** Saira Bashir, Abdul Haque, Yasra Sarwar, Aamir Ali, Muhammad Irfan Anwar

**Affiliations:** 1 National Institute for Biotechnology and Genetic Engineering (NIBGE), P.O. Box 577, Jhang Road, Faisalabad, Pakistan

**Keywords:** Uropathogenic *E.coli*, Phylogenicity, Virulence genes

## Abstract

**Background:**

Uropathogenic *E.coli* (UPEC) are among major pathogens causing urinary tract infections. Virulence factors are mainly responsible for the severity of these emerging infections. This study was planned to investigate the distribution of virulence genes and cytotoxic effects of UPEC isolates with reference to phylogenetic groups (B2, B1, D and A) to understand the presence and impact of virulence factors in the severity of infection in Faisalabad region of Pakistan.

**Methods:**

In this study phylogenetic analysis, virulence gene identification and cytotoxicity of 59 uropathogenic *E.coli* isolates obtained from non-hospitalized patients was studied.

**Results:**

Among 59 isolates, phylogenetic group B2 (50%) was most dominant followed by groups A, B1 (19% each) and D (12%). Isolates present in group D showed highest presence of virulence genes. The prevalence *hlyA* (37%) was highest followed by *sfaDE* (27%), *papC* (24%), *cnf1* (20%), *eaeA* (19%) and *afaBC3* (14%). Highly hemolytic and highly verotoxic isolates mainly belonged to group D and B2. We also found two isolates with simultaneous presence of three fimbrial adhesin genes present on *pap, afa*, and *sfa* operons. This has not been reported before and underlines the dynamic nature of these UPEC isolates.

**Conclusions:**

It was concluded that in local UPEC isolates from non-hospitalized patients, group B2 was more prevalent. However, group D isolates were most versatile as all were equipped with virulence genes and showed highest level of cytotoxicity.

## Background

Uropathogenic *E. coli* (UPEC) are among the major pathogens causing urinary tract infections (UTIs). Phylogenetic studies have revealed that these bacteria are not of very diverse origin and fall into four main groups (A, B1, B2, and D) [1,2] nowadays. Phylogenetic analysis is usually done by targeting three genetic markers *chuA*, *yjaA* genes and DNA fragment TSPE4.C2 [2]. This method has been found satisfactory and endorsed by other workers [3].

Virulent strains of *E. coli* mainly belong to phylogenetic groups B2 and D, while less virulent and commensal strains belong to phylogenetic groups B1 and A [2-7]. UPEC typically attach to epithelial cells with A-fimbrial adhesins (*afa*), P-fimbrial adhesins (*pap*), and S-fimbrial adhesins (*sfa*) [8-11]. Intimin, an attachment and membrane damaging pathogenicity factor, plays a vital role in virulence [12]. These virulence factors help bacteria to attach the epithelial cell lining of urinary tract and causes infection.

Hemolytic and cytotoxic necrotizing factors are also important virulence factors. The cnf1 is produced by 40% of uropathogenic *E. coli* isolates [13]. Most bacterial toxins cause initial cytopathic effects but only cytotoxins like cnf can lead to death of cultured cells [14]. In vitro alpha hemolysins and cytotoxic necrotizing factors have cytotoxicity towards different cells including Chinese hamster ovary, Vero and HeLa monolayers [15,16].

UTIs are very common in Pakistan and UPEC are the most frequent pathogens. Although a number of studies on this subject have been carried in various parts of the world, such data is not available from Pakistan. We felt that there was a need to characterize the local isolates of UPEC especially because no reports are currently available from this region.

## Materials and methods

### Collection, identification and storage of UPEC

Urine samples from non-hospitalized suspected cases of urinary tract infections were collected from various clinical laboratories of Faisalabad region. For isolation of *E. coli*, these samples were directly inoculated on MacConkey agar (Merck, Germany) plates. After overnight incubation at 37°C, lactose-fermenting colonies were identified as *E. coli* by characteristic morphology and inoculation on Triple Sugar Iron (TSI) agar (Merck, Germany) slants. The isolates were kept as stocks at −20°C in 50% glycerol stocks for long term storage.

### DNA Extraction and PCR based confirmation of *E. coli* isolates

Each isolate was inoculated in 5 ml of Tripticase Soy Broth (TSB). After overnight incubation at 37°C, DNA was extracted by conventional phenol chloroform method and quantified according to Sambrook *et al.*[17]. For confirmation of *E. coli,* PCR was performed by targeting *uidA* gene by following conditions described by Heninger *et al.*[18]. Primers are listed in Table 1.

**Table 1:** Oligonucleotide primers

**Genes**	**Primer sequences (5**^**/**^**to 3**^**/**^**)**	**Amplicon size (bp)**	**References**
*chuA*	GACGAACCAACGGTCAGGAT	279	[2]
	TGCCGCCAGTACCAAAGACA		
*yjaA*	TGAAGTGTCAGGAGACGCTG	211	[2]
	ATGGAGAATGCGTTCCTCAAC		
TspE4.C2	GAGTAATGTCGGGGCATTCA	152	[2]
	CGCGCCAACAAAGTATTACG		
*uidA*	ATCACCGTGGTGACGCATGTCGC	486	[18]
	CACCACGATGCCAT GTTCATCTGC		
*cnf1*	GGCGACAAATGCAGTATTGCTTGG	552	[19]
	GACGTTGGTTGCGGTAATTTTGGG		
*papC*	GACGGCTGTACTGCAGGGTGTGGCG	328	[20]
	ATATCCTTTCTGCAGGGATGCAATA		
*sfaDE*	CTCCGGAGAACTGGGTGCATCTTAC	408	[20]
	CGGAGGAGTAATTACAAACCTGGCA		
*afaBC3*	CATCAAGCTGTTTGTTCGTCCGCCG	793	[20]
	GCTGGGCAGCAAACTGATAACTCTC		
*eaeA*	GACCCGGCACAAGCATAAGC	384	[21]
	CCACCTGCAGCAACAAGAGG		

### Phylogenetic analysis

A triplex PCR was performed to segregate each isolate into one of the four main phylogenetic groups (A, B1, B2 and D) by targeting two marker genes (*chuA* and *yjaA)* and a DNA fragment TSPE4.C2 [2]. PCR conditions were same as described by Clermont *et al.*[2]. Primers are listed in Table 1.

### Targeted virulence related genes

There are many virulence genes associated with UPEC. We selected six most reported virulence genes. Among toxin encoding genes, *hlyA* gene was targeted for hemolysin and *cnf1* gene [19] for cytotoxic necrotizing factor. S-fimbrial adhesin is specific for terminal sialyl galactoside residues present on human erythrocytes; *sfaDE* gene [20] which is located on S-fimbrial gene cluster and is responsible for transport and assembling of fimbrial subunit was included, as well as *papC* gene [20] that is responsible for pyelonephritis associated adhesion, and *afaBC3* gene [20] that is located on *afa3* operon and is responsible for A-fimbrial adhesins. Yet another adhesin, intimin (encoded by *eaeA* gene) which has special significance in *E. coli* pathogenicity, was also included [21-23].

### Multiplex PCR for virulence related genes

Multiplex PCR was performed for each isolate for the identification of virulence related genes. Each 100μL multiplex PCR reaction mixture contained 1X PCR buffer (50 mM KCl, 10 mM Tris HCl; pH 8.3), 2.5 mM MgCl_2_, 100 nmol of each dNTP’s, 100 pmol of each primer, 5 U of recombinant *Taq polymerase,* (Fermentas, USA) and 2 μL of DNA template. Primers are listed in Table 1. The thermal cycler (MasterCycler; Eppondorf, Hamburg, Germany) conditions were as follows: 94°C for 5 min followed by 30 cycles of 94°C for 1 min, 56°C for 1 min and 72°C for 1 min and final extension of 5 min at 72°C. The PCR products were separated by electrophoresis on 2% (w/v) agarose gel and photographed by using Eagle Eye (Strategene, USA).

### Restriction analysis of virulence genes

PCR products of the virulence genes were subjected to restriction analysis for confirmation. The sequences of the genes were taken from NCBI GenBank using primer base sequences. The accession numbers of the genes are as follows: *papC* (**Y00529**), *sfaDE* (**X16664**), *afaBC3* (**X76688**), *hlyA* (**AB032930**), *eaeA* (**AF043226**), *cnf1* (**U42629**). These genes were restricted with HaeIII, Hinf1, Pst1, EcoR1 and Tail enzymes respectively. Restriction conditions were similar in all cases. Each 30 μL reaction mixture contained 1 μL of restriction enzyme, 8 μL of PCR product, 3 μL of specific enzyme buffer and 18 μL of sterilized dH_2_O. Overnight incubation was done at 37°C and the restriction products were separated on 2% agarose gel.

### Hemolysis assays

Hemolysin (*hlyA*) gene positive isolates were selected for studying hemolytic activity by using the method described by Haque *et al.*[24] with modifications. Human erythrocytes were used instead of rabbit erythrocytes and filtered supernatant of overnight culture of UPEC was double diluted with 0.9% NaCl in a 96 well microdilution plate. Rest of the procedure was as previously reported. Hemolytic unit was calculated by plotting dilution factor against transmittance. One hemolytic unit is the dose of hemolysin that causes 50% hemolysis.

### Vero cell lines and Toxicity assay

The cytotoxic effects of UPEC toxins (cytotoxic necrotizing factor and hemolysin) were studied by invading Vero cells. The Vero cell lines were grown in DMEM (MP Biomedical, USA), fetal calf serum 5-10%, gentamicin (1μL/mL) and amphotericin (10μL/mL) in sterilized Petri plates and incubated overnight at 37°C in the presence of 5% CO_2_. DMEM was removed from Vero cell plates followed by washing with PBS. Filtered and sterilized (0.2 μm pore size syringe filter) UPEC culture supernatant (1.5 mL) was added to each plate along with 1.5 mL DMEM and incubated overnight at 37°C with 5% CO_2._ The medium was removed from Petri plates into the falcon tubes. One mL of 1X trypsin: EDTA solution was added and after 5 min, dislodged cells were transferred to the same falcon tube. The falcon tube was centrifuged at 10,000 rpm for 10 minutes and supernatant was discarded; 1 mL of trypan blue was added, mixed gently and incubated at 37°C for 10 min. The ratio of dead (stained) and living cells was counted in a hemocytometer using inverted microscope (x4). The concentration resulting in 50% stained cells represented the 50% cytotoxic dose.

### Statistical analysis

Analysis of variance was used to compare the prevalence of various phylogenetic groups and presence of different virulence related genes. The difference having *P* value equal or less than 0.05 was considered as statistically significant.

## Results

### Confirmation of *E. coli* isolates

A total of 59 isolates were confirmed as *E. coli* by conventional biochemical identification followed by the detection of *uid*A gene.

### Phylogenetic analysis

Out of 59 UPEC isolates, the group B2 isolates 30 (50%) were found significantly higher (*P* < 0.05) as compared to other phylogenetic groups. Seven (12%) isolates fell in group D and 11 (19%) belonged to each of groups A and B1. Groups B1 and B2 showed two different patterns each. Details are given in Table 2.

**Table 2:** **Phylogenetic distribution of uropathogenic *E. coli***

**Phylogenetic groups**	**No. of UPEC isolate n = 59**	**Distribution according to gene groupings (n)**	***chuA*gene**	***yjaA*gene**	**TSPE4.C2**
Group B2	30 (50%)	28	+	+	+
		02	+	+	-
Group B1	11(19%)	09	-	-	+
		02	-	+	+
Group A	11(19%)	11	-	+	-
Group D	07 (12%)	07	+	-	+

### Prevalence of virulence genes among UPEC isolates

The targeted virulence genes were detected in 37 (63%) isolates. Remaining isolates were negative for the targeted genes. All the 7 group D isolates showed presence of these virulence genes. Breakup for other groups was: A 5/11 (45%), B1 6/11 (55%), and B2 18/30 (60%).

It was found that most prevalent gene was *hlyA* which was detected in 22 isolates (37%) while 16 (27%), 14 (24%), 11 (19%), 12 (20%), and 8 (14%) isolates were positive for *sfaDE, papC,eaeA, cnf1,* and *afaBC3* genes respectively. One isolate was positive for all 6 targeted virulence genes, two showed presence of 5 genes, and in three others 4 genes were detected. Three, two and one genes were found in 7, 10 and 14 isolates respectively. Detailed results are shown in Table 3.

**Table 3:** **Prevalence of virulence related genes in various phylogenetic groups of uropathogenic *E. coli *isolates**

**Phylogenetic groups**	**No. of Isolates with Virulence genes**	**Distribution of virulence genes**					
		***papC***	***sfaDE***	***afaBC3***	***eaeA***	***hlyA***	***cnf1***
Group B2	18/30 (60%)	10	08	04	05	11	05
Group B1	07/11 (64%)	02	03	01	01	02	03
Group A	05/11 (45%)	0	0	01	02	04	01
Group D	07/07 (100%)	02	05	02	03 05 03		
**Total**	**37/59 (63%)**	**14**	**16**	**08**	**11**	**22**	**12**

It was also found that *papC*, *cnf1* and *hlyA* were present collectively in 4 isolates, where as *papC*, *sfaDE* and *afaBC3* genes which represent P-fimbial, S-fimbrial and A-fimbrial adhesins respectively, were also identified collectively in two isolates as shown in the Table 4.

**Table 4:** Presence of virulence genes in UPEC isolates

**No. of Genes**	**Virulence Genes**	**No. of Isolates (n = 59)**
*6*	*sfaDE, afaBC3, papC, eaeA, cnf1, hlyA*	*1*
*5*	*sfaDE, papC, eaeA, cnf1, hlyA*	*1*
*5*	*sfaDE, afaBC3, papC, cnf1, hlyA*	*1*
*4*	*sfaDE, papC, eaeA, hlyA*	*2*
*4*	*afaBC3, papC, eaeA, hlyA*	*1*
*3*	*sfaDE, cnf1, hlyA*	*3*
*3*	*papC, eaeA, hlyA*	*1*
*3*	*afaBC3, eaeA, hlyA*	*1*
*3*	*afaBC3, papC, hlyA*	*1*
*3*	*papC, cnf1, hlyA*	*1*
*2*	*eaeA, hlyA*	*3*
*2*	*sfaDE, cnf1*	*1*
*2*	*sfaDE, hlyA*	*3*
*2*	*sfaDE,papC*	*2*
*2*	*papC,hlyA*	*1*
*1*	*sfaDE*	*2*
*1*	*afaBC3*	*3*
*1*	*papC*	*2*
*1*	*eaeA*	*1*
*1*	*cnf1*	*4*
*1*	*hlyA*	*2*
*0*	*No gene*	*22*

**Note:*sfaDE***, S-fimbrial adhesin; ***afaBC3***, A-fimbrial adhesin; ***papC***, P-fimbrial adhesin; ***eaeA***, Intimin; ***cnf1***, cytotoxic necrotizing factor; ***hlyA***, hemolysin.

The detected genes were confirmed by restriction analysis: *sfaDE* and *papC* amplicons (410 and 328 bp respectively) were restricted with HaeIII and yielded fragments of 209 bp, 135 bp and 66 bp; and 161 bp, 112 bp and 55 bp respectively. The *afaBC3* amplicon (793 bp) was restricted with Hinf1 and yielded fragments of 565 and 228 bp. The *eaeA* amplicon (384 bp) was restricted with EcoR1 and yielded fragments of 305 and 79 bp. Tail and msp1 were used to restrict amplicons of *cnf1* (552 bp) and *hlyA* (534 bp) genes respectively to yield fragments of 356, 192 and 4 bp, and 315 and 219 bp.

### Phylogenicity and virulence

When relationship of phylogenicity and virulence was studied, the prevalence of all virulence genes was found significantly higher in phylogenetic group D (*P* < 0.05) as compared to other phylogenetic groups except for *papC* gene which was found highest in group B2 isolates. The *papC* and *sfaDE* genes were absent in all group A isolates (Table 3).

### Hemolysis assay

Out of 59 isolates, 22 isolates were *hlyA* positive. All *hlyA* positive isolates (22) showed hemolytic activity in hemolysis assay. Hemolytic activity was classified into three groups, highly hemolytic (above 20 HU), moderately hemolytic (between 10–20 HU) and poorly hemolytic (below 10 HU). Accordingly, among 22 isolates, 3 (14%) were found highly hemolytic, 3 (14%) as moderately hemolytic and 16 (72%) as poorly hemolytic.

### Verotoxicity assay

Twenty seven isolates which were positive for *hlyA* and/or *cnf1* genes were selected for verotoxicity assay. Among 27 isolates, 15 were *hlyA* positive, 5 were *cnf1* positive and 7 possessed both genes. All the isolates showed verotoxicity. They were classified into highly toxic (80-100% cell death), moderately toxic (50-80%) and poorly toxic (below 50%). More details are given in Table 5.

**Table 5:** **Verotoxicity assay of *hlyA *and *cnf1 *gene positive UPEC isolates (n = 27) **

	***hlyA*gene positive isolates (n = 15)**	***cnf1*gene positive isolates (n = 5)**	***hlyA*** **+** ***cnf1*positive isolates (n = 7)**
Highly toxic	06	01	04
80-100% cell death			
Moderately toxic	05	03	01
50-80% cell death			
Poorly Toxic	04	01	02
Below 50% cell death			

## Discussion

UPEC are major cause of urinary tract infections and may be responsible for nearly 90% of UTI [25]. There are four main phylogenetic groups (A, B1, B2, and D) of UPEC and the phylogenicity has been reported to play an important role in virulence of these pathogens [1-3]. Although a number of studies on this subject have been carried in various parts of the world, such data is not available from Pakistan where UTI are very common. This study was designed to characterize local isolates of UPEC with respect to phylogenicity and distribution of most important virulence factors. Although our study is comprised of a relatively small number of samples and therefore faces limitations in statistical analysis, it provides important information about the phylogenetic background of uropathogenic *E.coli* from this region.

In the present study, we found that 50% of our isolates belonged to a single phylogenetic group B2 which is in line with some other studies [4,5].

We found that among all phylogenetic groups, most of the virulence related genes were present in significantly higher proportion in phylogenetic group D isolates except *papC* gene, which was more frequent in B2 isolates. This is in accordance with the report of Nowrouzian *et al.*[26] who found that most B2 strains carried genes for P-fimbriae.

Our results also supported some previous reports indicating greater association of traditionally recognized uropathogenic virulence factors (e.g. *pap*, *sfa*, and *hly*) with groups D and B2 as compared with A and B1 [6,27].

Among adhesins, *sfaDE* was highly (27%) prevalent among local UPEC isolates followed by *papC* (24%) and *afaBC3* (8%) genes. We found that frequency of *sfa* was higher as compared to other genes. In some of isolates, multiple adhesin genes were identified (*pap* and *sfa*; *pap* and *afa*; *pap*, *sfa* and *afa*) where as in some other isolates these genes were present independently. Here again, phylogenetic group D was most prominent. Our findings are comparable with those of Le Bouguenec *et al.*[20] who found *pap* operon in 79.4% of the pyelonephritis strains, either present alone (51.5%) or in association with either *afa* or *sfa* operon (27.9%), whereas in 12.4% and 22.7% of the isolates the adhesive properties were associated with the presence of *afa* and *sfa* operons, respectively. It is important to note that we did find these three operons in two isolates. The simultaneous presence of *pap*, *afa*, and *sfa* operons has not been reported before.

The intimin adhesin (*eaeA* gene) is an attachment and membrane damaging pathogenicity factor. It was found in 19% of the isolates. The presence of this adhesin gene has also been previously reported in UPEC [23] though it is well known that it is associated with diarrhoeagenic *E. coli*[21,28].

Among toxins, hemolysin was identified in 22 (37%) isolates. These findings are in line with those of Bingen-Bidois *et al.*[29] who found *hly* gene in 34% of UPEC isolates. In the present study, all *hlyA* gene positive isolates hemolysed human erythrocytes.

Our UPEC isolates showed considerable cytotoxic effects and 20% were positive for *cnf1* gene. When 27 (46%) UPEC isolates having *cnf1*, *hlyA* or both genes were used to invade the Vero cells, highest toxicity (80-100% vero cell death) was observed in *hlyA* gene positive isolates (n = 06), followed by *hlyA + cnf1* (n = 04) and *cnf1* positive (n = 01) isolates. This is in agreement with the reports suggesting that during the early stages of uropathogenesis, *hly* plays a major role in the damage of uroepithelium. It has also been reported that in many uropathogenic *E. coli* the loci of *hly* and *cnf1* are often linked and responsible for the severity of urinary tract infections [30].

High cytotoxicity levels were not always found associated with *cnf1* and *hly* positive isolates in this study because some isolates which were positive for other virulence genes, showed cytotoxic effect as well. On the other hand, some isolates with *cnf1* did not express any cytotoxic effect on Vero cells. It may be due to an insertion of a thymidine base at position 3325 in the C-terminal domain of *cnf1,* which encodes the catalytic region of the toxin [31,32]. The cumulative presence of *papC*, *cnf1* and *hlyA* is the evidence of pathogenicty island II_J96_, which is highly prevalent among UTI isolates [33]. In the present study the simultaneous presence of *papC*, *cnf1* and *hlyA* was observed in 4 UPEC isolates.

Highly hemolytic isolates belonged to phylogenetic group D, while highly cytotoxic isolates belonged to group B2 and D. This is in partial disagreement with the studies of Zhang *et al.*[34] who found that *hly* and *cnf1* positive isolates mainly belonged to phylogenetic group B2.

It can be concluded that all local UPEC isolates have a battery of virulence factors which makes them a serious and challenging health problem. The simultaneous presence of *pap*, *afa*, and *sfa* operons has been reported for the first time underlining the dynamic nature of these isolates. Another finding of interest was the prominence of Group D in relevance to the presence of virulence factors. The previous reports from other parts of the globe mostly highlight Group B2.

## Competing interests

All authors declare that they have no competing interest.

## Authors' contributions

SB carried out sample collection, analysis and drafted the manuscript, AH provided concept and finally drafted the manuscript, YS and AA provided guidance in Lab work, MIA helped in sample collection and preparation of manuscript. All authors read and approved the final manuscript.
